# Fine structure of mouthparts and feeding performance of *Pyrrhocoris sibiricus* Kuschakevich with remarks on the specialization of sensilla and stylets for seed feeding

**DOI:** 10.1371/journal.pone.0177209

**Published:** 2017-05-08

**Authors:** Yan Wang, Wu Dai

**Affiliations:** 1 State Key Laboratory of Crop Stress Biology for Arid Areas, College of Plant Protection, Northwest A&F University, Yangling, China; 2 Key Laboratory of Plant Protection Resources and Pest Integrated Management of the Ministry of Education, College of Plant Protection, Northwest A&F University, Yangling, China; Biocenter, Universität Würzburg, GERMANY

## Abstract

Mouthpart structure and feeding behavior in the temperate firebug, *Pyrrhocoris sibiricus* Kuschakevich, an important pest that feeds on seeds of leguminous and gramineous plants, are described for the first time. Mouthparts were observed using scanning electron microscopy to examine the external morphology, distribution and abundance of sensilla on mouthparts. Feeding performance by adults on both seeds and shoots were observed using a binocular microscope. The four-segmented labium contains 3 types of sensilla trichodea, 3 types of sensilla basiconica, 1 type of sensilla placodea and 1 type of sensilla campaniformia. Among them, sensilla trichodea are most abundant. The tripartite apex of the labium consists of two lateral lobes and an apical plate. Each lateral lobe possesses a field of 12 thick-walled uniporous peg sensilla and long non-porous hair sensilla. The mandibular stylet tips have three central teeth and two pairs of lateral teeth, which may help in penetrating hard seed coats. A series of scale-like projections are present on the inner surface of the mandibular stylets. The externally smooth maxillary stylets interlock to form a larger food canal and a smaller salivary canal, and there are five tubercles near the tip of the right stylet. Cross-sections of the stylet fascicle show that each mandibular stylet has a dendritic canal. The adult feeding process involves several steps, including exploring and puncturing of the host epidermis, a probing phase, an engorgement phase, and removal of the mouthparts from the host tissue. The structure and function of the mouthparts are adapted for the seed feeding habits.

## Introduction

Functional requirements linked with feeding have let to the evolution of a wide variety of morphological modifications of mouthparts that played a prominent role in the evolution of insects. Such modifications permit these invertebrates to feed upon nearly all organic materials [1]. Hemiptera, comprising the most successful radiation of hemimetabolous insects, show a wide array of ecological, behavioral, and morphological adaptations to a plethora of microhabitats and life-history strategies in virtually all ecosystems [2]. Plant-feeding hemipterans feed using their specialized piercing-sucking mouthparts to puncture tissue and to suck on leaf cell contents, vascular fluids or plant seed contents [3]. Variation in the morphology of mouthparts, especially the types of sensilla present, is correlated with variation in food source and feeding behavior [4,5]. The study of mouthparts can provide insight into feeding mechanisms [6,7] and may also provide important traits for use in assessing phylogenetic relationships [8].

Seed-sucking insects show morphological and physiological adaptations for exploiting seeds as a resource, particularly for piercing the often hard outer protective tissue of seeds before ingesting internal fluid [9]. The piercing-sucking mouthparts of seed-sucking insects have similar salivary ducts and are equipped with jigsawlike structures [3]. However, for these insects little is known about the finer aspects of mouthpart structure and their role in locating feeding sites within the host plant.

The hemipteran family Pyrrhocoridae is a small group comprising 33 genera and around 340 species worldwide [10,11]. Most Pyrrhocoridae feed on seeds or fruits particularly of plants belonging to the Malvales [12]. Previous studies on mouthparts in Pyrrhocoridae have mostly concentrated on terminal labial sensilla [6, 13–15], the interlocking mechanism of maxillae and mandibles of *Pyrrhocoris apterus* (L.) [16], and gross morphology of *Dydercus cingulatus* (F.) and *Odontopus nigricornis* (Stål) [7,17]. Other details on the mouthpart morphology of pyrrhocorid species have not been studied. The seed-bug, *Pyrrhocoris sibiricus* Kuschakevich, which is widely distributed in Russian Far East, Central and East Mongolia, China, North Korea and Japan, is a ground-dwelling polyphagous species that feeds on seeds of leguminous and gramineous plants [18, 19]. Both the nymphs and adults of *P*. *sibiricus* affect the health of plants primarily through the feeding on seeds. The fine structure of the mouthparts of *P*. *sibiricus* and the significance of mouthpart structures as they relate to various functions in feeding on seeds have not been previously studied.

Considerable information about feeding behavior of Hemipterans has been inferred from mouthpart structure [6,7], or was based on electropenetrograph (EPG) apparatus [20,21]. Consequently, ethological details about feeding behavior are lacking [22–24]. The images generated allow us to interpret the functional morphology of the component parts of the feeding apparatus and provide us with information to understand the actual feeding mechanism.

The aim of the present study was to conduct in-depth scanning electron microscope investigations to produce the first detailed morphological information on the mouthparts of *P*. *sibiricus* and to analyse details of feeding performance.

## Materials and methods

### Insect collecting

Adults of *P*. *sibiricus* used for SEM in this study were collected with sweep nets on *Hibiscus moscheutos* L. at the campus of Northwest A&F University in Yangling, Shaanxi Province, China (34°16′N, 108°07′E, elev. 563 m) in September 2015, preserved in 70% ethanol and stored at 4°C. For observing the performance of the mouthparts during feeding inside different types of substrates, additional adults of *P*. *sibiricus* were collected at the same locality in August 2016.

### Scanning electron microscopy

Adult males (n = 18) and females were placed in 70% ethanol and cleaned in an ultrasonic cleaner (KQ118, Kunshan, China) for 10s and rinsed with 70% ethanol several times. The heads were removed with dissecting needles under a stereomicroscope (Olympus SZX10, Japan) and then dehydrated in a series of successive ethanol solutions of 80%, 90% each for 20min and then dehydrated in baths of 100% ethanol twice, each for 30 min. Heads were then dehydrated in a graded series of tert-butyl alcohol (TBA) solutions of 25%, 50%, 75% (ethanol:TBA was 3:1; 1:1; 1:3) each for 15 min duration, and 100% TBA for 30 to 40 min duration. Then the samples were placed into a freeze -drier (VFD-21 S, SHINKKU VD, Japan) for 3 h. The dried specimens were mounted on aluminum stubs using double-sided copper sticky tape and coated with gold /palladium(40/60) in a high resolution sputter coater (MSP-1S, SHINKKU VD, Japan), and then examined with a Hitachi S-3400N SEM (Hitachi, Tokyo, Japan) operated at 15 kV.

### Feeding behavior on different types of substrates

To observe the feeding behavior of the mouthparts on dry seed and fresh twigs, some solid seeds and twigs of *H*. *moscheutos* L. were offered to twenty male and female individuals of *P*. *sibiricus* in an optical quality colorless glass 100 mm diameter, 135 mm tall. The insects were observed intermittently under a dissecting binocular microscope or through a headband magnifying glass throughout the feeding period for one week, and sequential images of adult feeding performance were taken using a Nikon D7000 camera when conditions were suitable. The images were saved directly to a computer for later analysis.

### Data analysis

The lengths of the mouthpart were compared between sexes using Student t-test. Statistical analyses were executed using SPSS 19.0 (SPSS, Chicago, IL).

### Image processing and terminology

Selected image files were analysed after being imported into Adobe Photoshop CS6 (Adobe Systems, San Jose, CA, USA). Measurements are given as means ± standard error of the mean. Schematic diagrams were drawn with Microsoft office Word 2016 and processed with Photoshop CS6. For classification of sensilla, the systems of Altner and Prillinger [25] were used in addition to the more specialized nomenclature from other studies [4,13,15,26–28].

## Results

### Gross morphology of mouthparts

**Fig 1:**
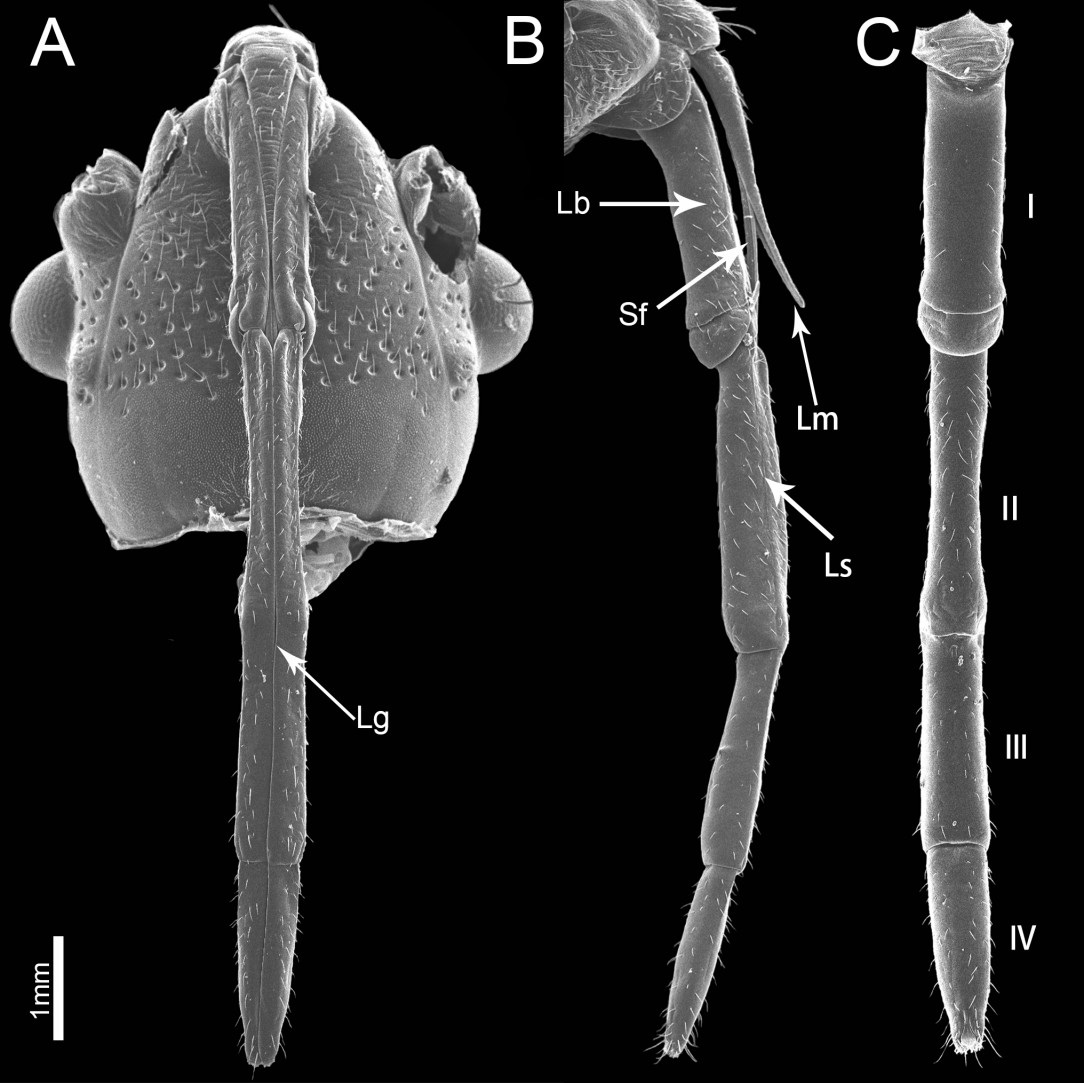
Scanning electron micrographs of the head of *Pyrrhocoris sibiricus*. (A) Anterior view. (B) Lateral view. (C) Dorsal view showing four-segmented labium (I–IV). Lg, labial groove; Sf, stylet fascicle; Lm, labrum; Lb, labium; Ls, longitudinal suture.

The piercing-sucking mouthparts of *P*. *sibiricus* resemble those of other heteropterans, arising from the front part of the head capsule and extending back along the ventral side of the body (Fig 1A). They are composed of the labium, labrum and a stylet fascicle consisting of two mandibular and two maxillary stylets (Fig 1B). The four-segmented labium has a labial groove located in the middle of the venter and is often curved dorsad when viewed laterally at rest (Fig 1A–1C). The two inner maxillary stylets are partially surrounded by two somewhat shorter and serrate-edged mandibular stylets which are housed inside the labial groove, proximally covered by the small cone-shaped labrum. There are different types of sensilla symmetrically arranged along the sides of the labial groove and on the apex of the surface of the labium. No obvious differences were noted between the mouthpart structure of females and males except for the length (t(8) = 4.473, P = 0.002). The total length in females is 3435.88 ± 90.79 μm (n = 7), and for males is 3169.93 ± 70.53 μm (n = 3).

### Labrum

**Fig 2:**
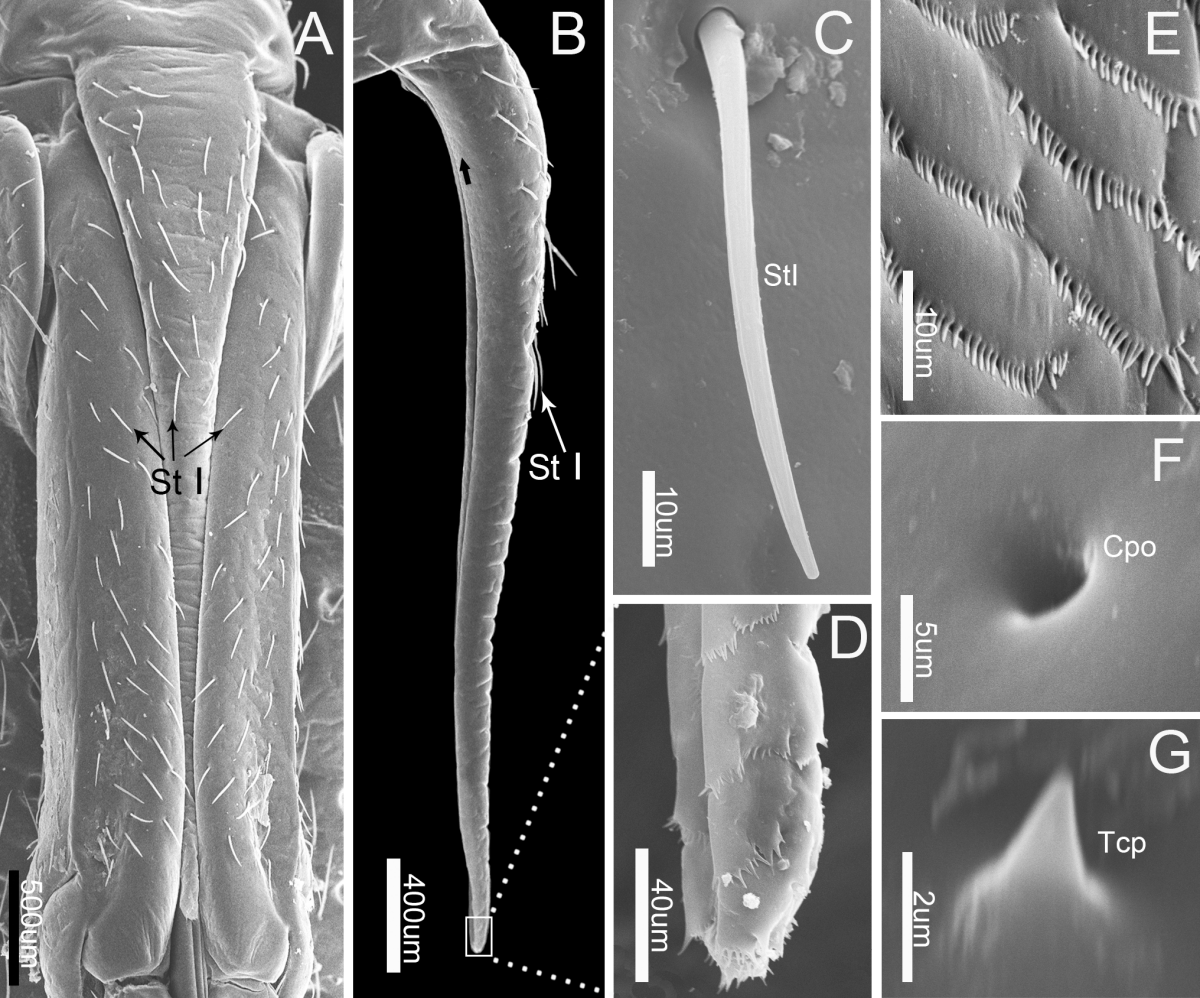
SEM of first labial segment and labrum of *Pyrrhocoris sibiricus*. (A) Anterior view of labrum and first labial segment. (B) Lateral view of labrum. (C) Enlarged view of sensilla trichodea I (St I). (D) Enlarged view of outlined box in (B), showing the tip of labrum. (E) Enlarged view of surface in (B) (black arrow). (F) Enlarged view of cuticular pores (Cpo). (G) Enlarged view of triquetrous cuticular processes (Tcp).

**Fig 3:**
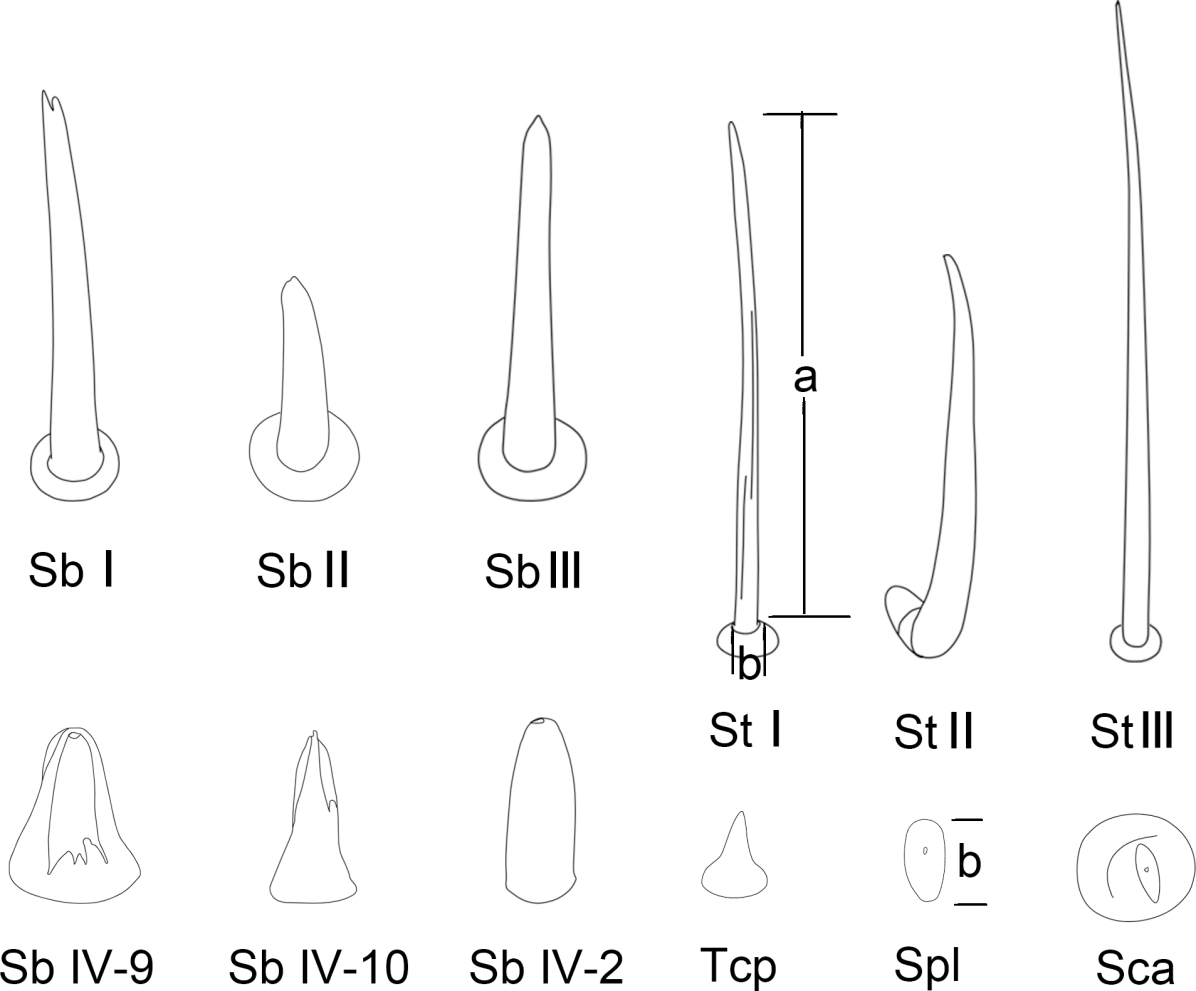
Diagrams of different types of sensilla and cuticular processes on mouthparts of female *Pyrrhocoris sibiricus*. St I, sensilla trichodea I; St II, sensilla trichodea II; St III, sensilla trichodea III; Sb I, sensilla basiconica I; Sb II, sensilla basiconica II; Sb III, sensilla basiconica III; Sb IV-2, No. 2 of sensilla basiconica IV; Sb IV-9, No. 9 of sensilla basiconica IV; Sb IV-10, No. 10 of sensilla basiconica IV; Sca, sensilla campaniformia; Spl, sensilla placodea; Tcp, triangular cuticular processes.

The cone-like labrum (899.67 ± 30.25 μm, n = 5), which is wider at the base and gradually tapers apically, is attached to the anterior edge of the anteclypeus and extends up to the proximal end of the first labial segment (Figs 1A, 1B and 2A). It is closely adpressed over the first labial segment and partly embedded in the labial groove (Figs 1A and 2A). The surface of the labrum is plicated and superficially traversed by numerous transverse grooves. Clusters of microtrichia are arranged in irregular transverse rows on the whole posterior area and the dorsal area of the labrum (Fig 2D and 2E) Sensilla trichodea I (Figs 2B, 2C and 3), which taper to the tip, triangular cuticular processes (Figs 2G and 3) and cuticular pores (Fig 2F) are also scattered on the labrum (Table 1).

**Table 1:** Morphometric data (mean ± SE) for various sensilla of adult *P*. *sibiricus* except for peg-like sensilla basiconica IV.

	Distribution	Length (μm)	Basal diameter (μm)	N
St I	Lm, Lb-sg1, sg2, sg3, sg4	47.36 ± 6.63	2.92 ± 0.40	20
St II	Lb-sg1	49.23 ± 9.4	3.24 ± 0.53	20
St III	Lb-sg4	94.39 ± 11.06	3.46 ± 0.39	20
Sb I	Lb-sg2, sg4	20.91 ± 5.59	3.79 ± 0.52	15
Sb I	Lb-sg2	7.21 ± 0.59	1.87 ± 0.22	15
Sb III	Lb-sg3	12.85 ± 2.48	1.97 ± 0.18	13
Sca	Lb-sg2	-	10.71 ± 2.32	18
Spl	Lb-sg4	-	3.58 ± 0.66	5
Tcp	Lm	1.01 ± 0.16	0.99 ± 0.14	4
Cpo	Lm, Lb-sg1	-	1.38 ± 0.33	20

N = sample size; Lm, labrum; Lb-sg1, the first segment of labium; Lb-sg2, the second segment of labium; Lb-sg3, the third segment of labium; Lb-sg4, the fourth segment of labium; St I, sensilla trichodea I; St II, sensilla trichodea II; St III, sensilla trichodea III; Sb I, sensilla basiconica I; Sb II, sensilla basiconica II; Sb III, sensilla basiconica III; Sca, sensilla campaniformia; Spl, sensilla placodea; Tcp, triquetrous triangular cuticular processes; Cpo, cuticular pores.

### Labium

The labium of *P*. *sibiricus*, a four-segmented tubular appendage, is suspended from the head capsule (Fig 1A–1C). When the insect is not feeding the labium lies between the coxae of the first and second pair of legs. The labium is bisected by a longitudinal groove extending the entire length and contains the stylet fascicle (Fig 1A). A longitudinal suture on each side form the lateral boundary of a cuticular strip separate from the lateral walls of the labium (Fig 1B). These sutures are indistinct in the first segment and reach only halfway to its tip of the last segment. There are several different types of sensilla generally distributed on each side of the labial groove. The tip of the labium has two lobes and an opening from which the stylet fascicle extends.

**Fig 4:**
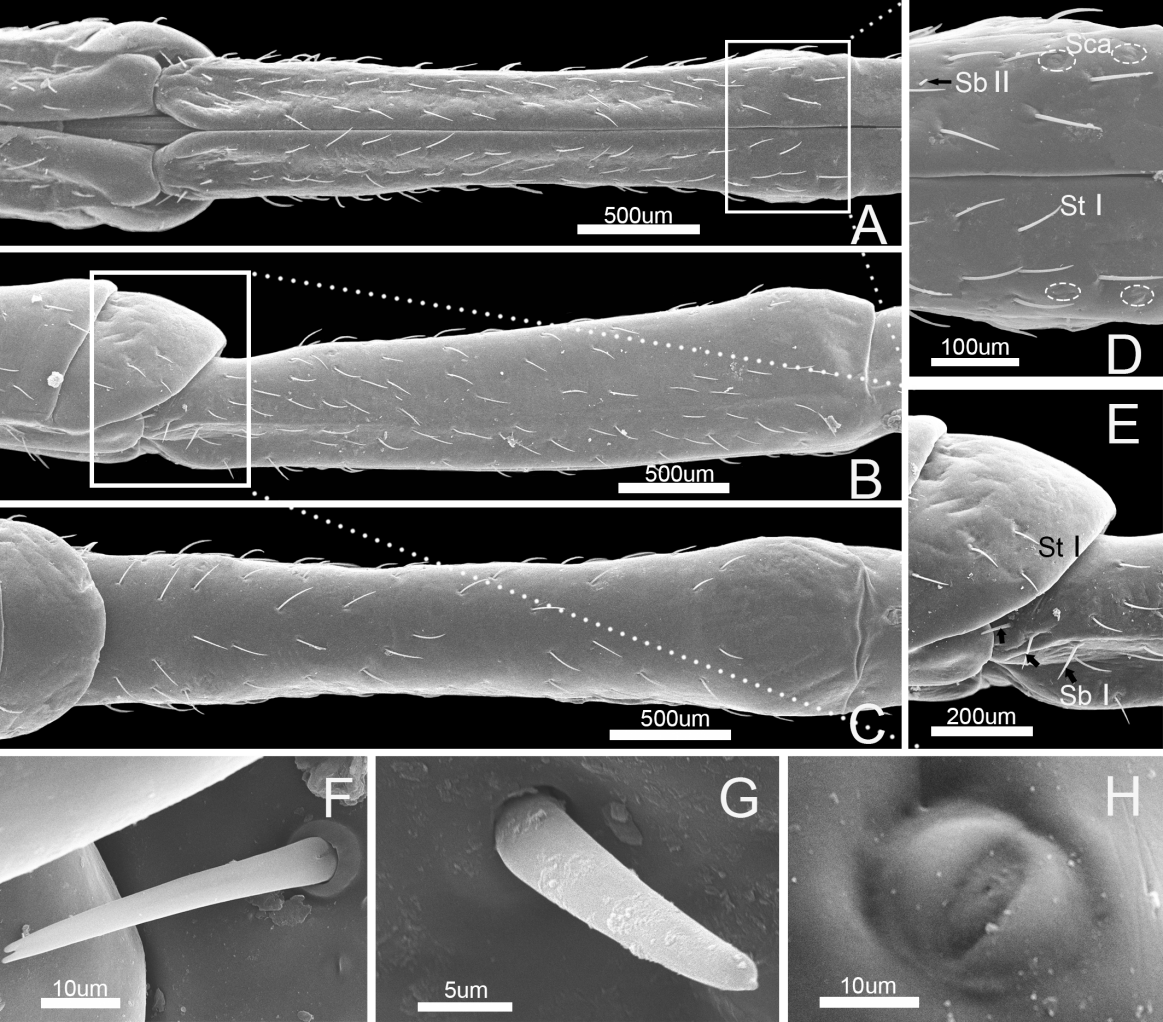
SEM of second labial segment of *Pyrrhocoris sibiricus*. (A) Anterior view. (B) Lateral view. (C) Dorsal view. (D) Enlarged view of outlined box of (A) showing sensilla trichodea I (St I), sensilla campaniformia (Sca) and sensilla basiconica II (Sb II). (E) Enlarged view of outlined box of (B) showing sensilla trichodea I (St I) and sensilla basiconica I (Sb I). (F) Enlarged view of sensilla basiconica I (Sb I). (G) Enlarged view of sensilla basiconica II (Sb II). (H) Enlarged view of sensilla campaniformia (Sca). Bars: (A), (B) and (C) = 500 μm; (D) = 100 μm; (E) = 200 μm; (F) = 10 μm; (G) = 5 μm; (H) = 10 μm.

The four labial segments differ in morphology and size. The proximal (first) segment is broad and of uniform width through most of its length with the distal part widened (Fig 2A, Table 2). A bandlike dorsal plate is present at the apex of the proximal segment, completely covering the joint between the two segments dorsally (Figs 1B, 1C, 4B and 4E). There are many sensilla trichodea I distributed on the sides of the labial groove and the lateral surface of the labium, but fewer sensilla on the posterior surface (Figs 1C and 2A). There are also many cuticular pores on this segment.

**Table 2:** Measurements of labrum, labium and stylets (mean ± SE) obtained from scanning electron microscopy.

		Length(μm)	Width(μm)	Height(μm)	N
Male	Lb	3169.93 ± 70.53			3
Female	Lm	899.67 ± 30.25	158.62 ± 6.76		5
	Lb-sg1	1041.93 ± 15.95	256.48 ± 10.75	223.28 ± 14.67	9
	Lb-sg2	994.04 ± 15.09	167.67 ± 9.29	168.46 ± 12.50	9
	Lb-sg3	779.92 ±33.62	202.85 ±10.66	166.02 ± 8.22	9
	Lb-sg4	663.29 ±19.67	182.86 ± 11.09	143.99 ±9.43	9
	Md	4083.18 ±348.78			10
	Mx	4203.59 ± 230.81			10

N = sample size. Lm, labrum; Lb, labium; Lb-sg1, first segment of labium; Lb-sg2, second segment of labium; Lb-sg3, third segment of labium; Lb-sg4, fourth segment of labium; Md, mandibular stylet; Mx, maxillary stylet.

The second segment is the longest and narrowest of the four segments, which is wide at the base, slightly narrowed to the middle and then widened to the end (Fig 4A–4C, Table 2). There is a tumid area at the end of the posterior surface of the second labial segment. Four types of sensilla were found on this segment, including three pairs of sensilla basiconica I that are arranged at the junction of the first and second segment (Fig 4B and 4E), many sensilla trichodea I that are arranged on each side of the labial groove and on the lateral surface and a few on posterior surface (Fig 4A–4D), two pairs of sensilla campaniformia that are arranged at the junction of the second and third segment (Fig 4D), and few sensilla basiconica II that are arranged on the anterior surface (Fig 4D). Sensilla basiconica I have a blunt tip and a minute longitudinal groove in the shaft, and insert in a cuticular sheath consisting of a cylindrical socket (Figs 3 and 4F, Table 1). Sensilla campaniformia have a convex, buttonlike central area surrounded by a ring of raised cuticle, with a small pore at the center (Figs 3 and 4H, Table 1). Sensilla basiconica II are conical, straight, robust and relatively short (Figs 3 and 4G, Table 1).

**Fig 5:**
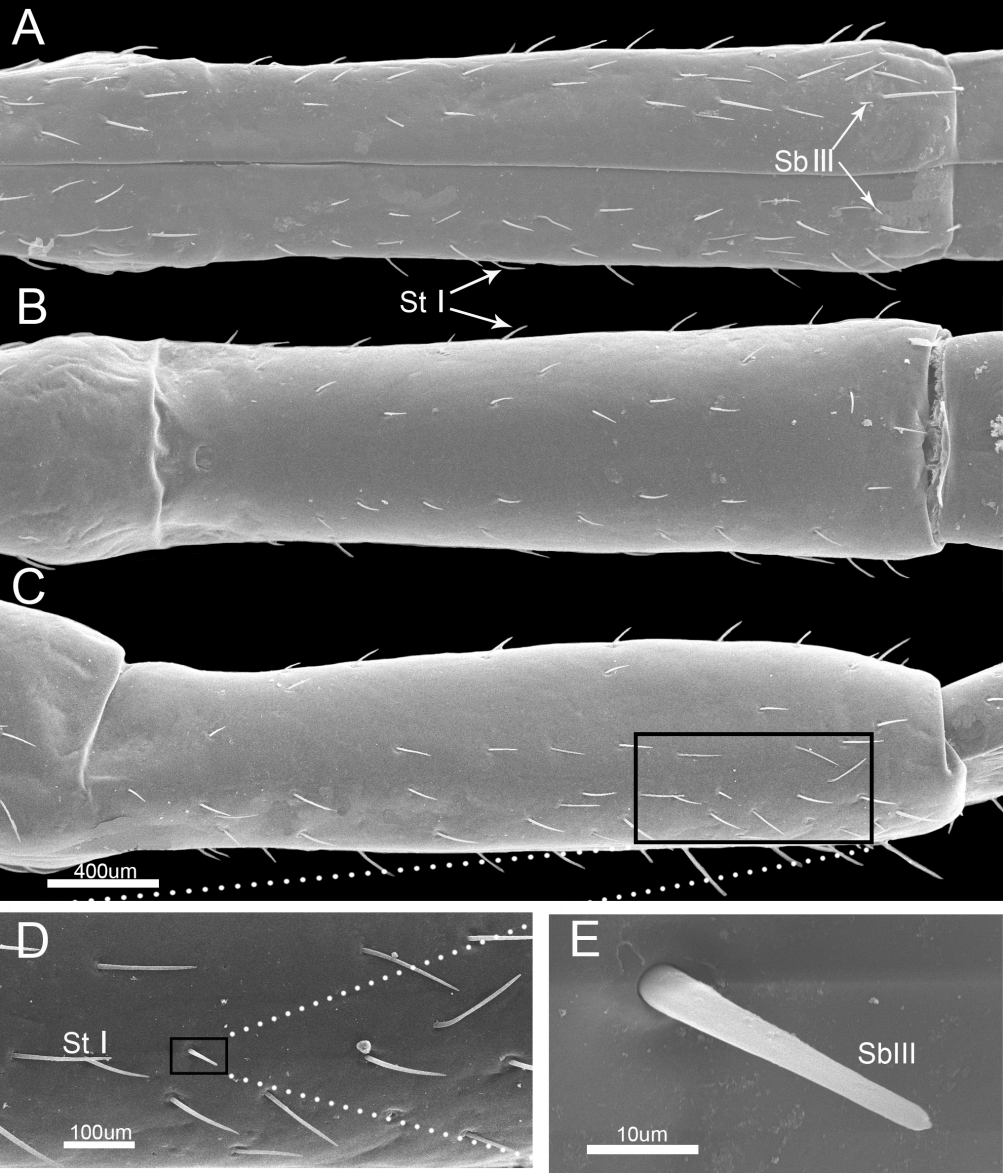
SEM of third labial segment of *Pyrrhocoris sibiricus*. (A) Anterior view. (B) Dorsal view. (C) Lateral view. (D) Enlarged view of outlined box in (C) showing sensilla trichodea I (StI). E. Enlarged view of outlined box in (D) showing sensilla basiconica III (SbIII). Bars: (A) (B) and (C) = 400 μm; (D) = 100 μm; (E) = 10 μm.

The third labial segment is shorter than the second segment and is broad and of nearly uniform width through most of its length (Fig 5A–5C, Table 2). It has many sensilla trichodea I and sparsely arranged sensilla basiconica III (Fig 5A–5D). Sensilla basiconica III are small pegs with tapered tips, arise from sunken pits and lack longitudinal grooves or apertures in the shaft (Figs 3 and 5E, Table 1).

**Fig 6:**
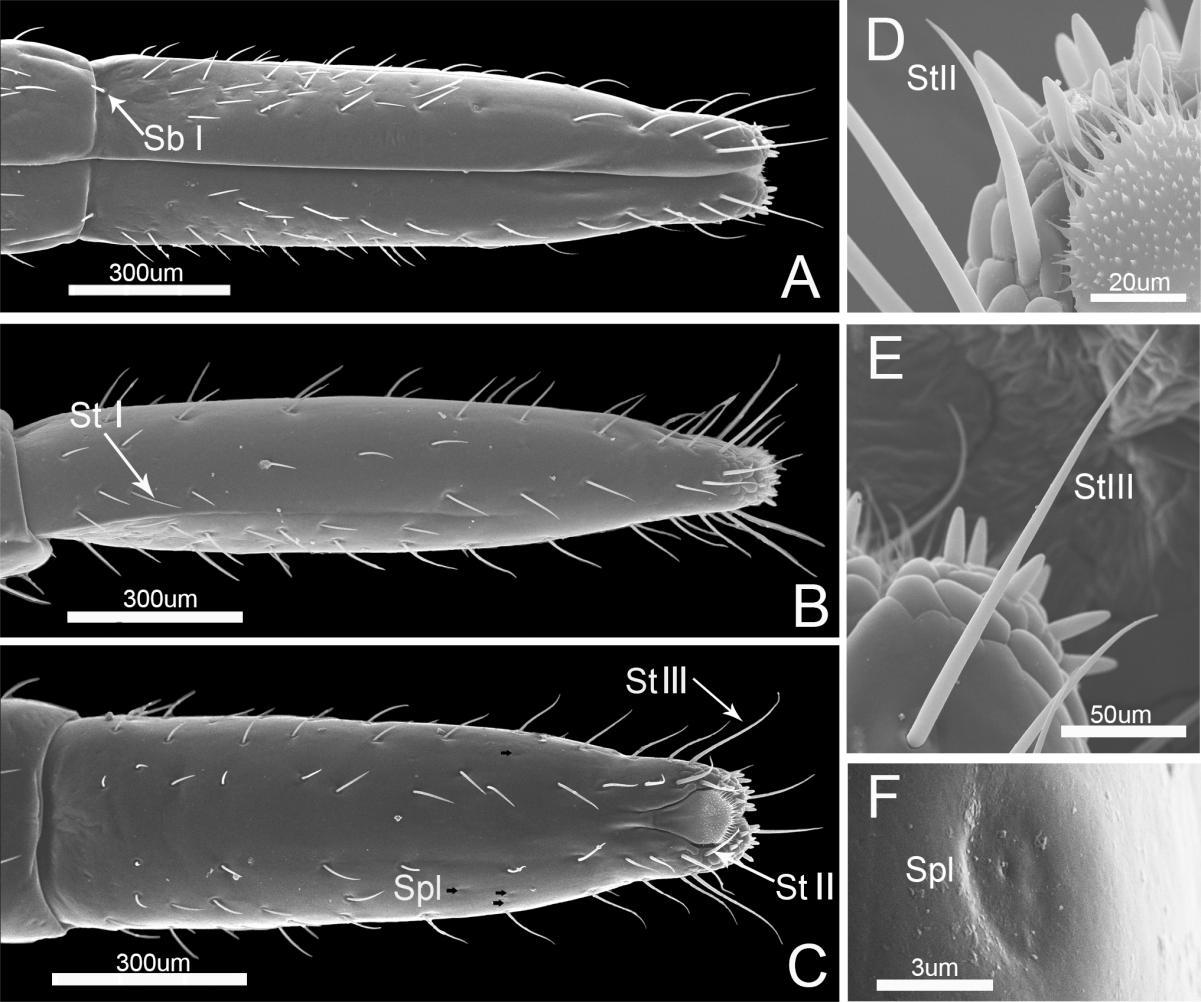
SEM of the fouth labial segment of *Pyrrhocoris sibiricus*. ***(***A) Anterior view. (B) Lateral view. (C) Dorsal view. (D) Enlarged view of sensilla trichodea II(StII). (E) Enlarged view of sensilla trichodea III(StIII). (F) Enlarged view of sensilla placodea (Spl).

The fourth, or distal labial segment is the shortest (Table 2). It is conical in shape and tapered distally (Fig 6A–6C). A large number of sensilla trichodea I are present laterally and dorsally but are absent on each side of the labial groove. Two sensilla basiconica I are present on each side of the junction of the third and fourth segments (Fig 6A). Six sensilla trichodea III, arranged roughly in a whorl encircling the labial subapex, are long and slender, slightly curved in the apical half, and inserted in sunken sockets (Figs 3, 6C and 6E, Table 1). Several sensilla placodea are located on the antero-lateral surface near the apical 1/3. Each is a sunken circular plate with a terminal pore (Figs 3, 6C and 6F, Table 1).

**Fig 7:**
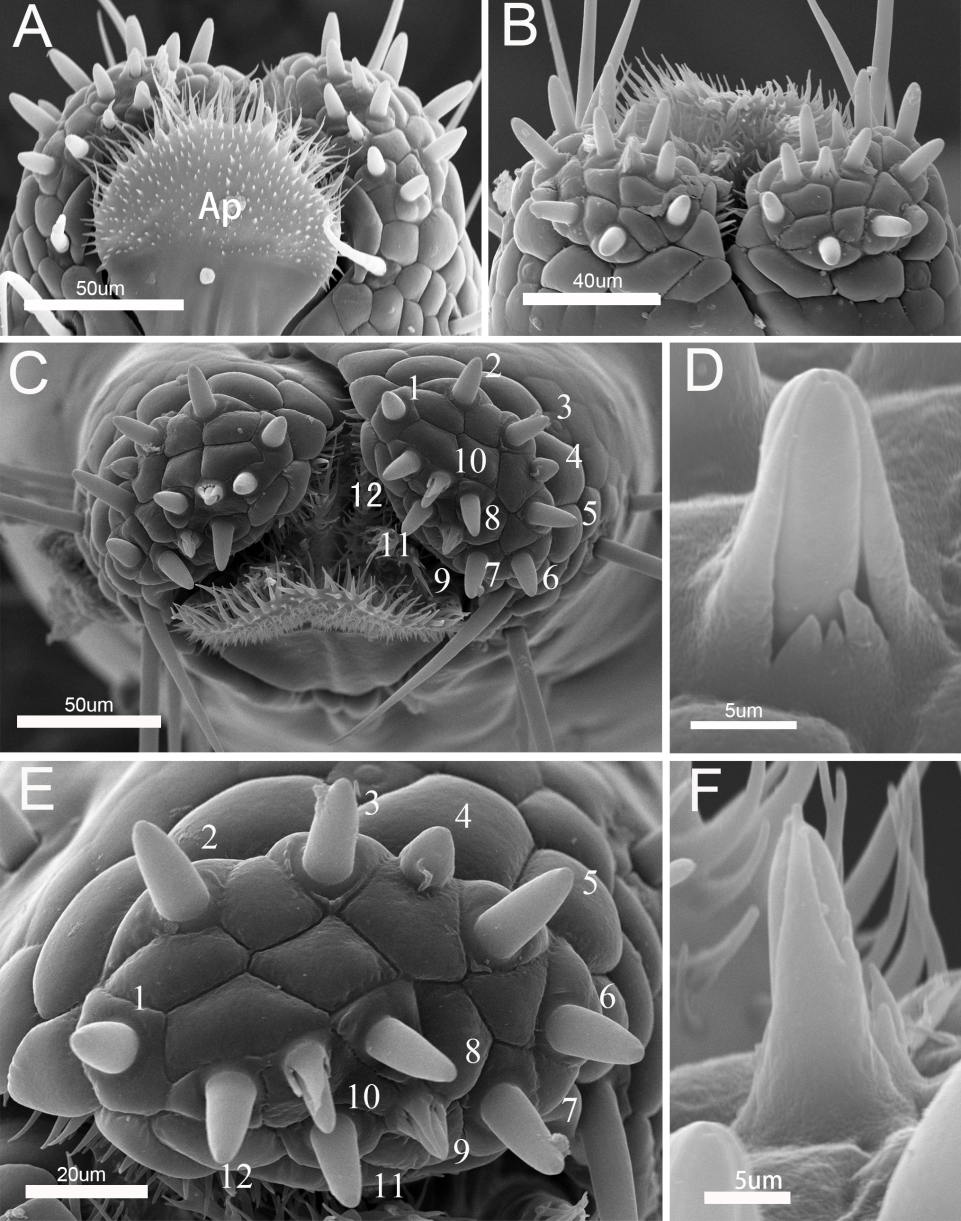
Distribution of sensilla on tip of labium of *Pyrrhocoris sibiricus*. A. Dorsal view of labial tip showing the cactoid apical plate (Ap); B. Anterior view of labial tip showing two lateral lobes; C. Vertical view of labial tip showing distribution of sensilla; D. Enlarged view of No.9 of sensilla basiconica IV; E. Enlarged view of right side of labial tip showing 12 sensilla basiconica IV; F. Enlarged view of No.10.of sensilla basiconica IV.

**Fig 8:**
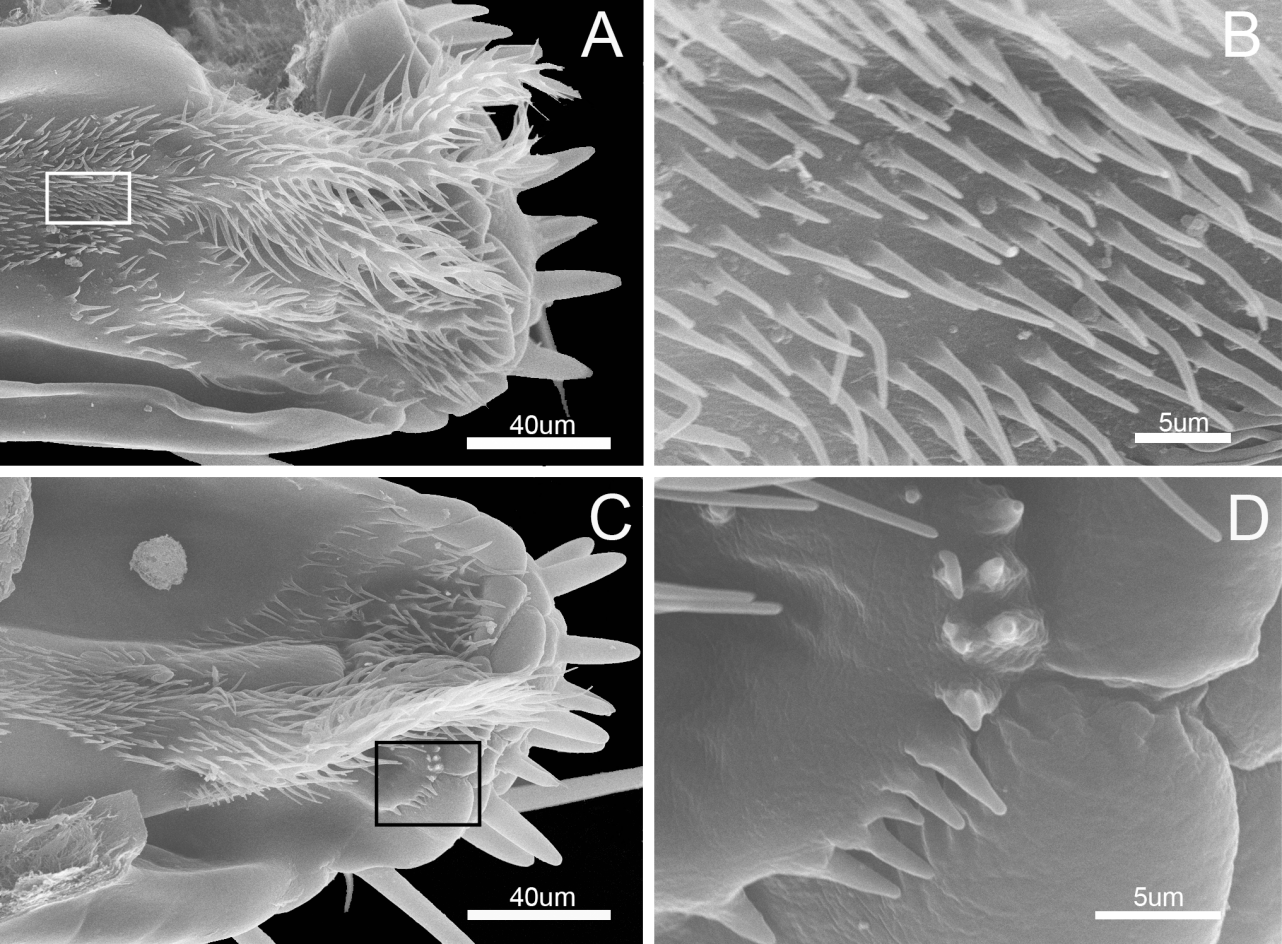
SEM of the inner surface of labium of *Pyrrhocoris sibiricus*. (A) Internal view. (B) Enlarged view of outlined box in (A) showing piliform cuticular processes. (C) Internal view. (D) Enlarged view of outlined box in (C) showing cuticular processes.

The labial tip is tripartite, distinctly divided into two lateral lobes and an apical plate (Fig 7A–7C). The cactoid apical plate is contained within the ventral groove and forms a rostral lid, covered ventrally by membranous microtrichia but apparently lacking sense-organs (Fig 7A). The lateral lobes consists of two sensory fields, including two main types of sensilla, long narrow sensilla trichodea II and short stout sensilla basiconica IV. A pair of long, pointed sensilla trichodea II is located on each side of the apical lobes behind the stylet groove. Twelve sensilla basiconica IV are present at the center of each lobe. The peglike sensilla basiconica are arranged in a central row (Fig 7C and 7E). The twelve sensilla basiconica vary in size and morphology (Table 3). They are tapered slightly from the base to the blunt tip, with sensilla 9 and 10 differing somewhat in that their tips become more pointed, both with an ovoid body, a socket surrounding the base of the shaft and a partial hood formed by fused cuticular processes (Figs 3, 7D and 7F). Sensilla trichodea II are hairlike with blunt tips (Figs 3 and 6D). All other pegs sit within a tightly fitting socket, and possess a single, terminal pore. Spines and comblike structures are laterally situated within the stylet groove of the last labial segment, probably serving to clean the mandibles during and after feeding (Fig 8A–8D).

**Table 3:** Mean±SE lengths (n = 15) of peg-like sensilla basiconica IV at labial tip of *P*. *sibiricus*.

No.	Shape	Length (μm)	Basal diameter (μm)	Tip
1	Stout	7.61 ± 2.31	3.9 7± 0.31	Blunt
2	Stout	8.68 ± 2.68	4.26 ± 0.19	Blunt
3	Stout	9.91 ± 0.55	3.8 9± 0.33	Blunt
4	Stout	6.29 ± 1.28	4.84 ± 0.68	Blunt
5	Stout	10.77 ± 5.49	4.81 ± 0.24	Blunt
6	Stout	8.31 ± 2.15	4.11 ± 0.15	Blunt
7	Stout	8.59 ± 3.09	3.60 ± 0.24	Blunt
8	Stout	8.28 ± 0.56	3.82 ± 0.23	Blunt, pore
9	Stout, perforated shaft	6.61 ± 0.94	3.55 ± 0.17	Blunt, pore
10	Stout, perforated shaft	5.94 ± 0.95	4.11 ± 0.36	Blunt
11	Stout	8.93 ± 2.71	3.86 ± 0.37	Blunt
12	Stout	6.67 ± 2.06	4.08 ± 0.32	Blunt

### Stylet fascicle

**Fig 9:**
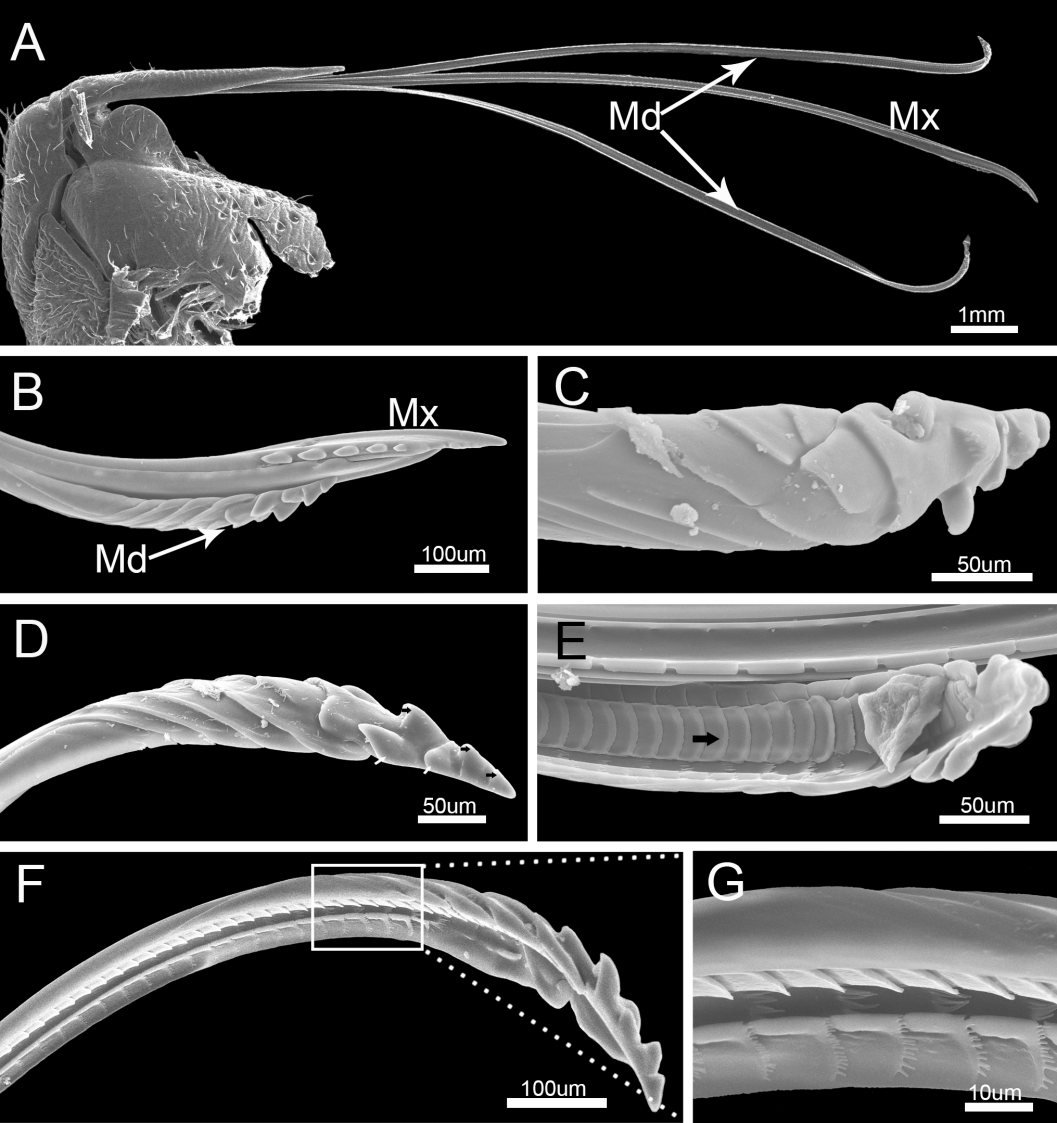
SEM of stylet fascicle and mandibular stylets of *Pyrrhocoris sibiricus*. (A) Stylet fascicle showing mandibular (Md) and maxillary stylets (Mx). (B) Anterior view of stylet fascicle. (C) External view. (D) Lateral view of mandibular stylet (Md) showing three central teeth (black arrow) and two pairs of lateral teeth (white arrow). (E) Interior view of mandibular stylet (Md) showing squamous texture (black arrow). (F) Lateral view. (G) Enlarged view of outlined box of (F).

The stylet fascicle is long, slender, and composed of two separated mandibular stylets (Md) and two interlocked maxillary stylets (Mx) (Fig 9A). Its dorsoventral and lateral axes are similar in diameter. The mandibular stylets (Md) are slightly shorter than the maxillary stylets (Mx) (Fig 9B, Table 2).

The mandibular stylets (Md) are adpressed laterally to the maxillary stylets (Mx). They are crescent-shaped in cross-section, convex externally and concave internally to form a groove for positioning of the maxillary stylets (Fig 9A and 9B). A regular series of transverse ridges is present on the outer surface of the mandibular stylet, including three central teeth and two pairs of lateral teeth near the apex(Fig 9B–9D). The dorsal surface has a row of serrate ridges and some scalelike projections are positioned on the lateral surface (Fig 9F). The inner surface of the mandibular stylet has a squamous texture, different from the dorsum and venter of the longitudinal groove (Fig 9E), that causes considerable friction against the outer surface of the adjacent maxillary stylet causing it to curve inward during probing of plant tissue.

**Fig 10:**
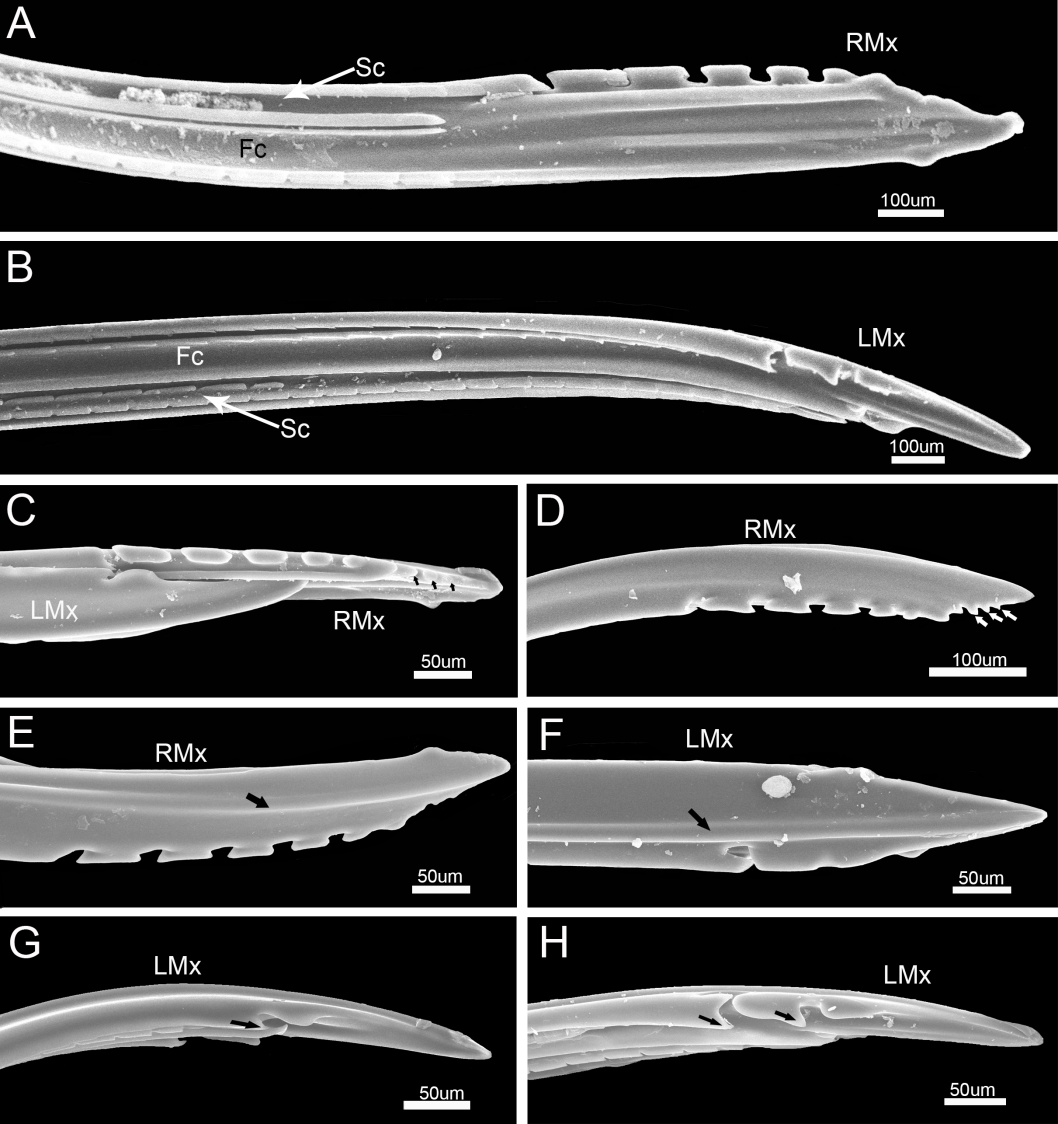
SEM of maxillary stylets of *Pyrrhocoris sibiricus*. (A) Apex of right maxillary stylet (RMx) showing food canal (Fc) and salivary canal (Sc). (B) Apex of left maxillary stylet (LMx) showing food canal (Fc) and salivary canal (Sc). (C) Apices of interlocked maxillary stylets showing inner surface of right maxillary stylet (RMx), outer surface of left maxillary stylet (LMx) and process (black arrow). (D) Apex of right maxillary stylet (RMx) showing process (white arrow). (E) Apex of right maxillary stylet (RMx). (F) External view of left maxillary stylet (LMx). (G) Lateral view of left maxillary stylet (LMx). (H) Lateral view of left maxillary stylet (LMx).

The maxillary stylets (Mx) are equipped with a series of ridges and grooves internally (Fig 10A and 10B) and a flange externally that engage grooves in the mandibular stylets. The distal end of each stylet is flattened and bears spines along the anterior and posterior margins on the external surface (Fig 10C–10H). These spines are arranged in two rows and are blunt and somewhat hooked, including five blunt spines (Fig 10C–10E) on the joint surface of the right stylet and two spines on the left stylet (Fig 10F–10H).

**Fig 11:**
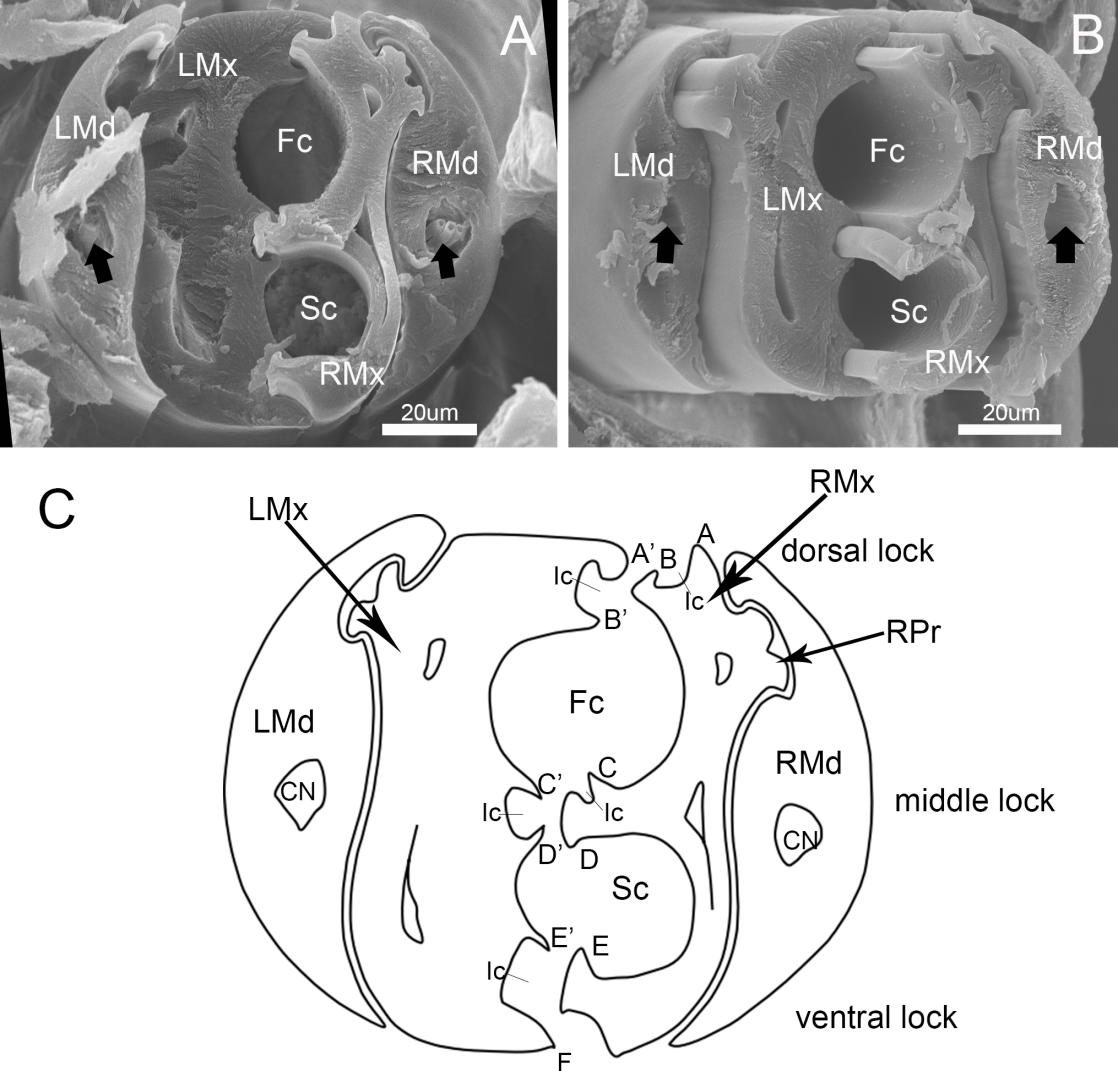
Cross-section of stylet fascicle of *Pyrrhocoris sibiricus*. ***(***A) and (B) Cross-section of stylet fascicle through middle of second segment and third segment showing shapes of mandibular stylets (Md), maxillary stylets (Mx), food canal (Fc), salivary canal (Sc) and dendritic canal (black arrow). (C) Diagram of cross-section of stylet fascicle. LMd, left mandibular stylet; RMd, right mandibular stylet; LMx, left maxillary stylet; RMx, right maxillary stylet; Fc, food canal; Sc, salivary canal; Ic, interlocking canal; CN, dendritic canal; RPr, Right process of the maxilla; A, Straight upper right process of dorsal lock; A’, Hooked upper left process of dorsal lock; B, Hooked lower right process of dorsal lock; B’, Straight lower left process of dorsal lock; C, Straight upper right process of middle lock; C’, Hooked upper left process of middle lock; D, T-shaped lower right process of middle lock; D’, Hooked lower left process of middle lock; E, Hooked lower right process of the ventral lock; E’, Hooked upper left process of ventral lock; F, Straight lower left process of ventral lock.

The left and right mandibular stylets are mirror images of each other in cross-section and each bears a dendritic canal located centrally in the thickest portion housing three dendrites that run the length of the stylet (Fig 11A–11C). The two asymmetrical maxillary stylets are interlocked by hook-like hinges (Fig 11A–11C), including five processes on the arm of the right maxilla and six on the left (Fig 11C). On the left maxilla, the dorsal and ventral locks are formed by two processes: a hooked upper one and a straight lower one. The middle lock is formed by two hooked processes (Fig 11A–11C). On the right maxilla, the dorsal lock is formed by a straight process and a hooked process. The middle lock consists of essentially the same mechanism as the dorsal lock, but its lower process is T-shaped (Fig 11A–11C). The ventral lock is only formed by a hooked process. The interlocked maxillary stylets are taller than wide in cross-section (Fig 11C), and form a salivary canal (Sc) that delivers saliva to the plant, and a food canal (Fc) which is used to suck plant fluids. The hollow food canal (11.74 ± 0.96 μm, n = 8) is ovoid in cross-section and its diameter is slightly greater than that of the salivary canal (8.66 ± 0.25 μm, n = 8), which is circular in cross-section and situated mostly in the right maxilla. On the internal extreme end of the stylets, the salivary canal joins the food canal to deliver a small portion of secreted saliva to digest plant fluids (Fig 10A and 10B). The maxillae are slender and sharply pointed at the tips. Within each mandibular stylet there is one approximately semicircular dendritic canal (Fig 11A and 11B).

### Feeding of *P*. *sibiricus* on plant seeds and shoots

**Fig 12:**
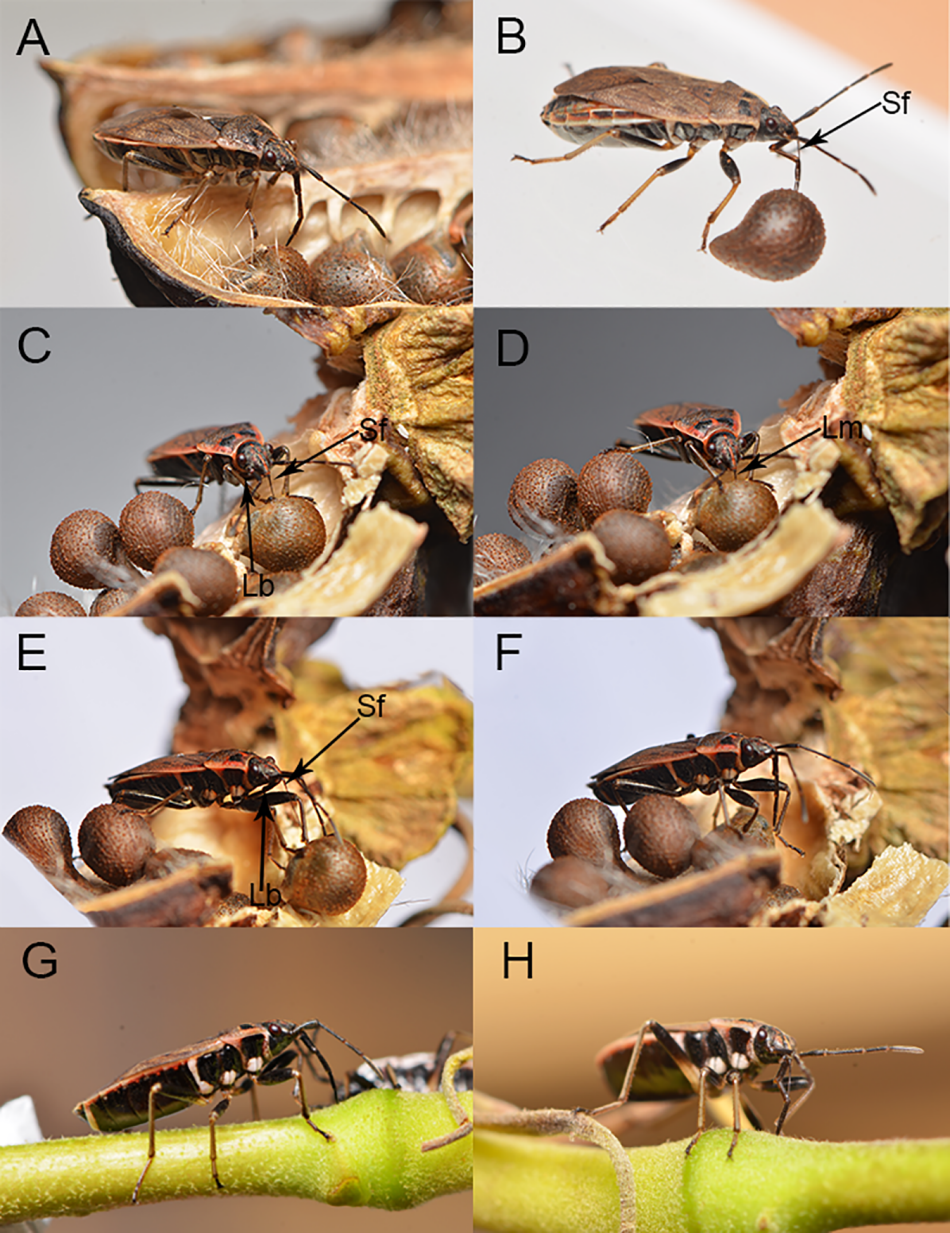
Feeding stages on mature seeds and young stalks in adult *Pyrrhocoris sibiricus* showing positions of the mouthparts. **(**A) Location of suitable feeding position by the labium. (B) Puncture of seed by stylet fascicle showing elbow-like fold of proximal and second rostral segments and stylet penetration. (C) Start of feeding showing puncture of epidermis by stylets and labium withdrawn below the ventral side of the head and thorax. (D) Feeding on seed. (E) Termination of feeding showing retraction of stylets. (F) Use of forelegs to return stylet fascicle to labial groove. (G) penetration of green stalk. (H) Feeding on green stalk. Lm, labrum; Lb, labium; Sf, stylet fascicle.

Images of adult feeding movements were captured as viewed from the side when feeding on plant seeds (Fig 12A–12F) and their parts (Fig 12G and 12H). Data from all such images are summarized in the following description of feeding kinematics.

The adult feeding process involves several steps, including exploring and puncturing of the host epidermis, a probing phase, an engorgement phase, and removal of the mouthparts from the host tissue. These processes vary slightly in mouthpart position and time.

To initiate feeding, *P*. *sibiricus* first chooses a suitable position on the host surface by probing with sensilla at the tip of the labium. The apical segment of the labium is oriented vertically on the feeding surface, anchored in place and the stylets are inserted into the substrate (Fig 12A).

When a dry or fresh seed was offered to the insects, they use their mandibles (serrated structures borne by the mouthparts) to puncture the seed coat and insert the long, flexible stylet fascicle. Usually the stylets were inserted through the micropyle of the seed or through cracks, if any, in the hull. If the hull was free of cracks, individuals used their mouthparts to pierce the hull. During this process, the first and second segments elbow toward the insect’s body shortening the functional length of the labium and allowing the stylets to penetrate deeply (Fig 12B). If penetration is successful, the labium continues to bend to a maximum angle at which angle between the second segment and the first segment is less than 90 degrees (Fig 12B). The stylets in the bundle continue to slide back and forth against each other while the labium folds back below the ventral side of the head and thorax (Fig 12C). When the head capsule is near the host surface (Fig 12D), the labrum pulls away from the stylet bundle allowing the stylet to penetrate deeper. While the stylets are inserted, the stylets fascicle alternately penetrates and is retracted from the seed again and again. When the stylet fascicle is withdrawn from the surface of the seed (Fig 12E), the labium bends forward away from the insect body and beside the stylet fascicle, and the front legs are used to re-insert the fascicle into the labial groove (Fig 12F). Finally, the labium rotates back to its normal resting position beneath the ventral surface of the body.

The process of feeding on young shoots of the plant is similar to that observed for seed feeding except that the stylets are never fully retracted from the labium (Fig 12G and 12H).

## Discussion

Insect feeding encompasses the search for a host organism, location of a suitable feeding site, penetration of host tissue with the mouthparts, ingestion and digestion. The mouthparts have evolved in ways that allow insects to effectively exploit their food sources, and the structure of mouthparts reflects the type of diet utilized and feeding habits. Convergent evolution in the various taxa of fluid-feeding insects has led to various kinds of piercing-sucking mouthparts. Because of the important role of Hemiptera in agriculture and human disease transmission, the piercing -sucking mouthparts have been extensively studied in many hemipteran families [16,29–36]. The mouthparts of different hemipterans have evolved slight functional differences. Compared with other families, the mouthparts of the Pyrrhocoridae adults appear to display a number of traits that are apparently derived within Heteroptera [29].

As far as we know, this is the first study to investigate in detail the mouthparts of Pyrrhocoridae at the fine-structural level, and is the first comprehensive description of the stylet fascicle and labium of this family. The mouthparts of *P*. *sibiricus* are highly similar, though not identical, to those of another pyrrhocorid, *O*. *nigricornis* (Stål) in general structure [6] and to those of *O*. *nigricornis* Stål [6] and *Dysdercus fasciatus* (Signoret) [13], *Dysdercus intermedius* Distant [15], *Dysdercus fulvoniger* (De Geer) and *Dysdercus koenigii* (F.) [14] in the structure and arrangement of labial tip sensilla.

Various traits of the stylets including the shape and dentition of the tips and size of the food canal have been studied previously in several heteropterans [29,37,38]. However, in Pyrrhocoridae, the stylets have been poorly described compared to sensory receptors on the labium. The mandibular stylets of *P*. *sibiricus* surround the maxillary stylets and have three central teeth and two paired lateral teeth on the distal extremity, as well as five or six oblique parallel ridges on subapex of external convex region. Similar structure is also found in other heteropteran species but the numbers of teeth are different. As in *O*. *nigricornis* Stål, the teeth in *P*. *sibiricus* are relatively prominent and stout, but they differ from those of other heteropteran in number and pattern [15,29,32,33,38,39]. The sharp ends of the mandibular stylet are specialized to pierce plant tissues while probing. The tooth-like protrusions on the side of the mandibular stylets are also used to stabilize the maxillary stylets during probing and anchor the stylet fascicle in host tissues, serving as a fulcrum for the movement of the maxillae [15,29,32,38,40]. The inner surface of the mandible has a complex ribbed scalelike texture that causes considerable friction against the outer surface of the adjacent maxillary stylet [29].

Maxillary stylets are asymmetrical only in the positions of internal longitudinal carinae and grooves. Their inner surfaces show traces of small, widely spaced notches arranged in longitudinal strips. Our observations of cross sections through the tips of the maxillary stylets show that the interlocking mechanisms of *P*. *sibiricus* is similar to those of Fulgoroidea [41], Coccinea [42], Cixiidae [43] and Peloridiidae [44]. The internal structure of *P*. *sibiricus* mouthparts revealed that both maxillary and mandibular stylets are flattened laterally, thus they are taller than wide in cross-section [16]. The salivary canal is in the right maxilla, with the left serving only as a closing strcuture [16].

Different types of cuticular sensory receptors occur on various areas of the labium to discriminate complex chemical and mechanical stimuli that are produced by the host. High sensillar diversity and /or abundance on the labium have been observed in other hemipterans, and these apparently perform both chemosensory and mechanosensory functions, during probing with the labium during plant surface exploration [4,6,13,15, 29–31,36,45–49]. Detailed morphological descriptions of pyrrhocorid mouthpart sensilla have never been reported. The labium of *P*. *sibiricus* is equipped with seven types of sensilla on the tip and surface which are likely mechanoreceptory and chemosensory.The labial tip, which always contacts the host surface during the host selection and feeding, usually has poreless mechanosensory hairs and uniporous or multiporous pegs [44]. Many previous workers described rostral sensillae and their possible function as chemoreceptors and mechanoreceptors [4,6,7,50–52]. We observed two types of sensilla on the tip of the labium of *P*. *sibiricus*, sensilla trichodea II and sensilla basiconica IV. Sensilla trichodea with flexible sockets in the subapical region of the labium in this species appear to be identical to those of other heteropterans [6,13,31,46,47], in which the structure indicates a contact chemoreceptive function. Twenty-four sensilla basiconica are found in two sensory fields, as reported in the pyrrhocorids *D*. *intermedius* Distant [15], *D*. *fulvoniger* (De Geer) and *D*. *koenigii* (F.) [14] but differing from the ten sensilla basiconica in *O*. *nigricornis* Stål [6] and *D*. *fasciatus* (Signoret) [13]. Other pentatomorphans vary in the number of sensilla, e.g., 16 and 18 sensilla basiconica in the alydid *Riptortus pedestris* F. and the lygaeid *Elasmolomus sordidus* (F.) respectively [7]; 22 sensilla basiconica in *N*. *viridula* L. [6], *Blissus leucopterus leucopterus* (Say) [53] and *Lygus lineolaris* (Palisot de Beauvois) [46, 47]. Generally, the positions and arrangement of sensilla were similar among the studied phytophagous species, and sensilla basiconica on the apical sensorial region probably have contact chemoreceptor—gustatory functions.

Not all sensilla on the labium may be used to contact and evaluate potential food. The pair of sensilla basiconica I on the junction between the first and second segment, and the third and fourth segment, which also occur in *Cacopsylla chinensis* (Yang et Li) and *Eriosoma lanigerum* (Hausmann), may function as proprioceptors to perceive the degree of flexion of the joint [27,28,54–56]. Sensilla basiconica Ⅱ are arranged on the anterior surface of the third segment and are similar in appearance to those described by Brożek and Bourgoin [4] and Chen [57]. Yet, the function of these sensilla is not clear. Sensilla basiconica III with bare tips found on the fourth labial segments of the insects examined are similar in appearance to the sensilla described in *O*. *nigricornis* Stål and *N*. *viridula* L. [6]; and in *R*. *pedestris* F., *E*. *sordidus* (F.), *Cyclopelta siccifolia* Westwood and *Chrysocoris purpurea* (Westwood) [7]. It appears that this type of sensilla is common in most Heteroptera, but their function is not clear.

Sensilla campaniformia (SCa) occur on various body regions of insects, including mouthparts, antennae, bases of wings, halteres, legs, and eyes [26,58–60]. Two pairs of campaniform sensilla are arranged bilaterally at the distal part of the anterior surface of the second labial segment of *P*. *sibiricus*. Similar sensilla in Peiratinae (Reduviidae) have been shown to have a proprioreceptory function [48] and such mechanosensory sensilla also occur in phytophagous Pentatominae and predatory Asopinae (Pentatomidae) [31]. In pyrrhocorids, such sensilla probably act as proprioceptors responding to the stresses arising from the movement of the labium.

The structure of the apical plate suggests that it does not function in any sensory capacity. Cobben [29] also presents evidence that the apical plate of several Hemiptera has no sensory function. According to Hatfield and Frazier [46], the internal structure of the apical plate, including its cuticular projections, consists of layered cuticle throughout its length and lacks neurons and other organized cellular structures. Cobben [29] suggested that the salivary flange is supported by the apical plate; its presence is correlated with the serrated maxillae, and it is possibly involved in providing support, allowing the stylet bundle to be extended apically in a specific lateral orientation.

A dorsal plate is like a stretch-bandage at the apex of the proximal segment, completely covering the joint between the two terminal segments dorsally. It is here termed the elbow plate, following Rathore [17] who described such a structure in *D*. *cingulatus* (F.). This configuration must limit the lifting potential of the proximal segment and probably provides better control over the movements of the rostrum (allowing the rostral segments to be compressed dorso-ventrally when they "elbow" during feeding, shortening the functional length so that the stylets may penetrate deep into the food source) between the first and the second segment (Fig 12B).

In summary, this study has revealed new information about the mouthparts of *P*. *sibiricus*, including some details of the fine structure not previously observed in Heteroptera. This will contribute to a better understanding of the sensory system and feeding behavior of seed-sucking bugs. Additional study will be needed to determine the function of some of the structures observed in *P*. *sibiricus*.
